# The Global Potato-Processing Industry: A Review of Production, Products, Quality and Sustainability

**DOI:** 10.3390/foods14101758

**Published:** 2025-05-15

**Authors:** Xiaoye Hu, Hong Jiang, Zixuan Liu, Mingjie Gao, Gang Liu, Shilong Tian, Fankui Zeng

**Affiliations:** 1 Lanzhou Institute of Chemical Physics, Chinese Academy of Sciences, Lanzhou 730000, China; 2 University of Chinese Academy of Sciences, Beijing 100049, China; 3 State Key Laboratory of Efficient Utilization of Arid and Semi-Arid Arable Land in Northern China, Institute of Agricultural Resources and Regional Planning, Chinese Academy of Agricultural Sciences, Beijing 100081, China; 4 Agricultural Product Storage and Processing Research Institute, Gansu Academy of Agricultural Sciences, Lanzhou 730070, China

**Keywords:** potato processing, product analysis, tech challenges, industry trends

## Abstract

The global potato industry has changed dramatically over the past half century—the potato-planting area in Poland decreased from 2,819,200 hectares in 1961 to 188,580 hectares in 2023, representing a 1394.96% relative decrease; South Africa’s potato production increased from 332,000 tons in 1961 to 2.42 million tons in 2023, representing a 627.60% relative increase. This study provides a comprehensive comparison of the potato-processing industries in China and major global producers. The global potato-processing market was valued at USD 40.97 billion in 2023 and is projected to reach USD 60.08 billion by 2031, with significant variations in production and consumption patterns across countries. As the world’s largest potato producer, China processes approximately 15% of its total potato output, whereas India, the second-largest producer, processes only about 7%. In contrast, developed countries such as the United States, Canada, and leading European nations—including Germany, the Netherlands, France, and Belgium—demonstrate significantly higher levels of processing, underpinned by advanced technologies, automation, and efficient quality-control systems. In order to conduct an in-depth analysis of the competitiveness of China’s potato-processing industry, this paper employs the Diamond Model to carry out relevant research. Despite rapid progress, China’s potato-processing industry still lags behind these global leaders in key aspects such as automation, production efficiency, and product quality. Differences remain evident in major processed potato products, including French fries, potato chips, potato flakes, and starch, as well as in raw-material supply chains, environmental sustainability, and market competitiveness. However, China’s role in the global potato-processing industry is evolving. A major milestone was reached in 2022 when China became a net exporter of frozen French fries for the first time, signaling a shift in its position in the international market. This transformation highlights China’s emergence as a key player in global French fry exports and suggests a potential restructuring of the industry. While challenges remain, the growing acceptance of Chinese French fries in international markets reflects improving product quality. Future industry trends point toward increased automation, product innovation, circular economy practices, and greater international market integration. To enhance its competitiveness, China must further modernize its processing industry, adopt cutting-edge technologies, strengthen quality control, and expand its global footprint to secure a stronger position in the evolving international potato-processing landscape.

## 1. Introduction

Potato (*Solanum tuberosum* L.) is one of the world’s most important food crops, playing a crucial role in global food security due to its wide adaptability and high nutritional value [1,2]. As a key raw material in the food industry, potatoes can be processed into a diverse range of products, including French fries, potato chips, potato flour, and starch [3]. These products are in high demand on international markets and are increasingly expanding into the health food and novel food sectors [4].

Compared to the international market, China’s potato-processing industry began later. However, in recent years, driven by policy support and market demand, the industry has experienced rapid development [5]. For instance, in August 2022, Snowvalley Liupanshan Food (Ningxia) Co., Ltd. successfully launched a new production line with a capacity of 20 tons per hour for frozen French fries, with products exported to over thirty countries worldwide. By 2024, the company had also completed testing and launched a 25,000 ton annual production line for potato flakes. These milestones reflect the increasing scale and technological advancement of China’s potato-processing industry. In parallel, China’s frozen French fry exports have grown steadily, making the country a net exporter for the first time in 2022. In the first ten months of 2024 alone, China’s French fry exports reached 155,100 tons, highlighting its shifting role in the global French fry market [6]. China is emerging as a key player in global French fry exports, potentially contributing to a periodic restructuring of the industry. Leading Chinese potato-processing companies are expanding their international presence, with 40% of Kaida Hengye’s revenue coming from overseas markets, while Snowvalley’s frozen French fries are now exported to over thirty countries, including those in Southeast Asia, Japan, South Korea, Australia, New Zealand, Fiji, and parts of South America.

The contribution of technological advancements to the development of the potato industry cannot be overlooked. In 2021, the contribution of generalized technological progress to China’s potato yield was as high as 59.25%. With the combined forces of technological progress, policy support, technological advancements, infrastructure development, international collaboration, and market demand, the evolution of China’s potato-processing industry has been positively precipitated in recent years. However, compared to developed countries in Europe and North America, China still faces notable challenges, such as insufficient processing technology, low levels of equipment localization, and limited product diversity [7].

To gain a clearer understanding of the development status and future directions of China’s potato-processing industry, this paper provides a comparative analysis of the market size, processing technology, product innovation, and development trends in both China and leading global producers. The paper comprehensively examines the challenges and opportunities faced by China’s potato-processing industry.

## 2. Materials and Methods

Obtain basic data such as the planting area, production volume, and trade volume of major global potato-producing countries in 2023 and earlier from the FAOSTAT database (https://www.fao.org/faostat/en/#data/QCL, accessed on 5 December 2024). Use the United Nations Commodity Trade Database (https://comtrade.un.org/, accessed on 5 December 2024) to collect the import and export data of potatoes and related products in various countries, covering multiple commodity categories such as fresh or chilled potatoes, potato flakes, and potato starch. At the same time, refer to the data released by official institutions such as the United States Department of Agriculture (USDA) and Agriculture and Agri-Food Canada to supplement and verify the accuracy of the acquired information.

Collect industry reports released by institutions such as Data Bridge Market Research and Statista to obtain data on the global potato-processing market size, growth trends, and market shares of major enterprises. Refer to the reports issued by food industry associations, such as the reports of the Potato Food Professional Committee of the China National Food Industry Association and the Potato Starch Branch of the China Starch Industry Association, to obtain detailed data on China’s potato-processing industry, including information such as the production volume of various products and the production capacity of enterprises.

Select representative countries such as the United States, Canada, India, and Germany as cases to deeply analyze the development models of their potato-processing industries. By comparing the market sizes of different countries and based on the progress of some countries in product innovation and processing technology, this paper analyzes China’s position and development potential in the global potato-processing market.

In this study, we have cited data from multiple statistical agencies to analyze the global and Chinese potato-processing industries. Given that different statistical agencies may vary in their definitions and statistical methods for key concepts such as “potato production” and “processed potatoes”, a unified explanation is provided here to avoid misunderstandings. The “potato production” statistics in the FAOSTAT database refer to the total amount of potatoes used for various purposes after harvest, covering aspects such as food consumption, feed, processing, and losses. However, in the actual statistical process, there may be slight differences in the statistical scopes of different countries. Some countries may have certain errors in the statistics of the portion of potatoes retained by farmers for their own use. The potato production statistics by the United States Department of Agriculture (USDA) focus on the quantity harvested from commercial farms and entering the market circulation and processing sectors, and do not fully cover the portion used by farmers for their own consumption. When it comes to the statistics of processed potatoes, it mainly refers to the amount of potatoes consumed in the production of industrial food products such as French fries, potato chips, and starch. In the Ministry of Agriculture and Rural Affairs of China statistics’ domestic potato-related data, the potato production includes the portion used by farmers for their own consumption and the portion in the market circulation. However, regarding the statistics of the market scale of the processing industry, it mainly covers the economic value of large-scale processing enterprises, and the statistics of small workshops or individual processing may not be comprehensive enough. When comparing the data of different countries, we have made every effort to consider the impact of these differences on the analysis results. Nevertheless, due to the diversity of data sources and the complexity of statistical methods, there may be certain limitations in the data comparison. We hope that readers will fully consider these factors when referring to the data of this study, so as to more accurately understand the global potato-processing industry landscape and the development status of the Chinese industry.

## 3. Current Status of Global Potato-Processing Industry

This chapter mainly expounds on the current status of the global potato-processing industry from aspects such as planting area, output, processing ratio, market scale, and trade situation, aiming to present the status and differences in different countries in this industry and provide a basis for subsequent comparative analysis.

The FAOSTAT database (https://www.fao.org, accessed on 11 November 2024), updated at the end of December 2024, provides potato production statistics up to 2023 [8]. In 2023, the total global potato harvest area was 16.79 million hectares, slightly higher than the previous year.

Table 1 offers a comparative analysis of potato production, harvest area, and processing proportion in 2023 across 11 major potato-producing countries, namely China, the United States, Canada, India, Germany, Poland, the Netherlands, France, the United Kingdom, Belgium, and South Africa [8]. These data not only reflect the diversity of the potato industry in these countries but also highlight the distinct production and consumption patterns across different regions.

In terms of potato production, the Food and Agriculture Organization (FAO) defines it as the total yield of potatoes harvested for both human consumption and other uses, including seed and feed. China and India stand out as the largest producers, with outputs of 93,491,819 tons and 60,142,000 tons, respectively. Their substantial production volumes contribute significantly to the global potato supply, indicating their dominance in potato cultivation. Other countries like the United States (19,992,090 tons), Germany (11,607,300 tons), and France (8,606,490 tons) also play crucial roles in the global market, each with their unique production characteristics.

The harvest area data reveal variations in potato cultivation scales. China has a relatively large harvest area of 4,571,531 hectares, providing a vast foundation for its high production. In contrast, some European countries such as the Netherlands (155,340 hectares) and Belgium (95,700 hectares) have smaller areas but often adopt intensive cultivation methods, leveraging advanced agricultural techniques to achieve high yields per hectare.

Processed potatoes typically refers to potatoes that have undergone some form of industrial processing, such as freezing, dehydration, or the use of potato-related products. When it comes to the processing proportion, significant differences exist among countries. Developed countries like the United States, Canada, and Germany exhibit high processing rates. The United States processes 64.46% of its total potato production, with a large portion going into the production of frozen French fries and other processed products, catering to the demands of the fast-food industry. Canada, with a processing proportion of 68%, mainly focuses on processing potatoes into fries, chips, and other products. Germany, where potato processing accounts for 70–80% of total production, excels in starch production and the manufacturing of advanced potato-based products.

On the other hand, India and China have relatively lower processing proportions. India processes only about 7% of its potato output, suggesting a large untapped potential for its potato-processing industry. China, with a processing proportion of approximately 15%, has room for growth in converting its abundant potato production into value-added processed products. These lower processing rates may be attributed to differences in food culture, market demands, and the level of industrial development.

The global potato-processing market was valued at USD 40.97 billion in 2023 and is expected to grow to USD 60.08 billion by 2031, exhibiting a compound annual growth rate (CAGR) of 4.90% from 2024 to 2031, with the majority of the market concentrated in North America, Europe, and Asia [9]. Frozen and snack foods such as French fries and potato chips are in high demand globally, particularly driven by the rapid growth of the fast-food industry. Data from Statista shows that the global potato-processing market was valued at around $32 billion in 2019, with an expected increase to $51.1 billion by 2030 [10].

Table 2 presents a comparison of the export (EXP) and import (IMP) volumes of five major potato-related commodities across 11 key potato-producing countries in 2023 (UN Comtrade 2023) [11]. These commodities include fresh or chilled potatoes (070190), potato flakes, granules, and pellets (110520), potato starch (110813), frozen potatoes prepared or preserved through methods other than by vinegar or aceic acid (200410), and non-frozen potatoes prepared or preserved through methods other than by vinegar or aceic acid (200520). The trade volumes of these commodities in terms of both imports and exports reflect the diverse role potatoes play in global agricultural trade, highlighting the varying production and consumption patterns in different regions.

Fresh or chilled potatoes (070190) show substantial trade flows, with some countries acting as major exporters and others as significant importers. For example, countries such as France, Germany, and the Netherlands are prominent exporters, leveraging their advanced agricultural practices and infrastructure to supply fresh potatoes to global markets. In contrast, the Netherlands, despite being large producers, exhibit the highest import volumes, suggesting domestic consumption outpaces local production or a preference for specific varieties not grown domestically.

Potato flakes, granules, and pellets (110520) are highly processed forms of potatoes and exhibit more complex trade patterns. Export leaders include Germany, the Netherlands, the United States, and Belgium, where the value-added processing of potatoes has been a well-established industry. On the import side, the United States and Germany are notable for their high demand for processed potato products, likely due to their rapidly growing convenience food industries.

Potato starch (110813) trade is typically dominated by countries with highly developed agricultural processing sectors. Germany and the Netherlands feature as significant exporters, capitalizing on their strong potato-processing industries. Conversely, the United States remains the largest importer, reflecting its substantial demand for starch in various food and industrial applications. The trade of potato starch is indicative of the global supply chain’s integration, where producing nations also benefit from processing and exporting this versatile product.

Frozen potatoes prepared or preserved other than by vinegar or aceic acid (200410) as a key convenience food show a mixed trade pattern. Belgium, the Netherlands, Canada, and the United States lead in exports, leveraging their developed food-processing industries to meet global demand, particularly from fast-food chains and retail markets. On the import side, countries like the United States, the United Kingdom, and France show significant volumes. These imports highlight the demand for ready-to-eat or easy-to-prepare potato products in these regions.

Non-frozen potatoes prepared or preserved other than by vinegar or aceic acid (200520), including canned or otherwise preserved potatoes, display similar trade dynamics to frozen potato-processing products (200410) but with slightly less global movement. The Netherlands, Belgium, and the United States are key exporters, while countries like Germany and France exhibit moderate levels of imports. The lower export and import figures suggest that the preservation methods used (excluding vinegar or acetic acid) are more localized in their appeal, with varying demand across regions.

The data for 2023 underscore the significant role of both fresh and processed potato products in global trade. While countries like the United States, Canada, Netherlands, Belgium, and Germany dominate as exporters, driven by their well-established processing industries, China and South Africa continue to be major importers of processed potato products, highlighting differences in consumption patterns and the need for specific types of potato products not available domestically. The trade flows reflect broader global trends, such as increasing urbanization, the growing demand for convenience foods, and the expanding global supply chain for potato-based commodities.

## 4. Comparison Between Major Producers

### 4.1. United States

In the United States, a significant portion of potatoes is used for French fry production [12]. The U.S. is a major exporter of frozen French fries, shipping substantial quantities to markets worldwide [13], including Japan, South Korea, Mexico, China, the Philippines, and Thailand. However, due to the pressures of global supply chains and rising domestic demand, U.S. imports of French fries have also increased, primarily from Canada and several European countries.

**Figure 1:**
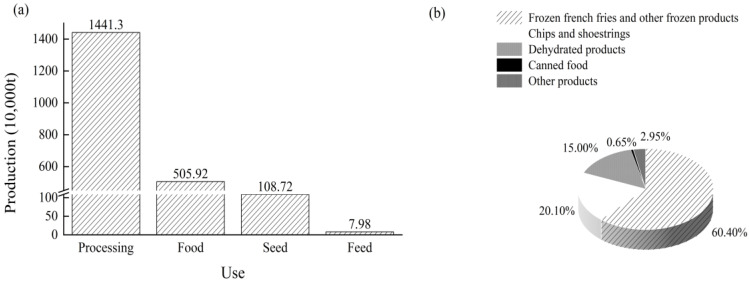
The distribution of potato utilization in the United States in 2023 (**a**); the proportions of various potato-processing methods in the United States in 2023 (**b**).

In 2023, the United States used approximately 5 million tons of potatoes for fresh food consumption. The total U.S. potato production in 2023 was 22 million tons (adapted from USDA), with 14.41 million tons processed, representing 64.46% of total production (Figure 1a). The largest share of processing was allocated to frozen French fries and other frozen products, accounting for approximately 62.03%. The next largest share was for chips and shoestrings (20.37%), followed by dehydrated products (15.05%), canned food (0.65%), and other products (2.96%) (Figure 1b).

### 4.2. Canada

**Figure 2:**
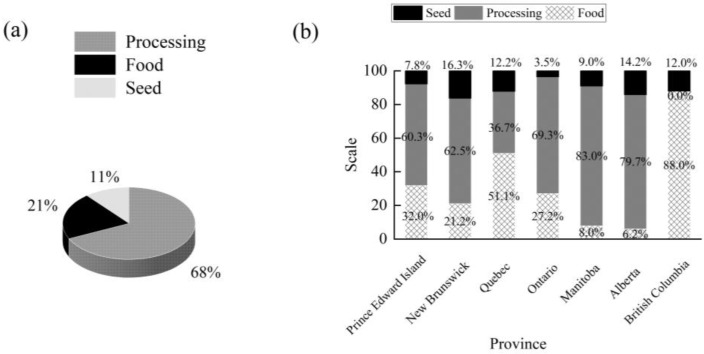
Distribution of potato utilization in Canada in 2023 (**a**); distribution of potato utilization in different provinces of Canada in 2023 (**b**).

Potatoes are the largest vegetable crop grown in Canada, with three primary uses: seed, food, and processing [14]. In 2022, Canada’s potato consumption structure showed that 2.763 million tons of potatoes were used for fresh consumption, while 240,000 tons were allocated to seed consumption. In 2023, approximately 68% of Canada’s total potato production was used for processing, 21% for fresh consumption, and 11% for seeds (Figure 2a). The total potato production in 2023 was about 5.8 million tons (adapted from Agriculture and Agri-Food Canada) The majority of processed potatoes are used to produce French fries, potato chips, and other potato products [15].

The main provinces for French fry processing in Canada, based on production volume, include Alberta, Manitoba, New Brunswick, and Prince Edward Island, while Ontario has the largest sector for potato chips (Figure 2b). Processing plants are generally located near production areas. These processors typically enter into contracts with potato growers ahead of the growing season to ensure the required quantities and varieties of potatoes [16]. Most growers have advanced storage facilities and are responsible for maintaining high-quality potatoes to meet the specifications of retailers and processors.

Canadian potato varieties, such as Snowden, are prized for their excellent quality and moderate starch content, which enhances their competitiveness in international markets, further driving export demand [17]. The food-processing industry is a key area of potato demand in Canada [14]. As the population grows, the demand for potatoes as a food source remains steady [18]. Major fast-food chains, such as McDonald’s, have a steady and sustained demand for French fries and other potato products in their numerous outlets across Canada, further stimulating the domestic market.

### 4.3. India

India, as the second-largest potato producer in the world, has a relatively low processing ratio of only 7%, which contrasts sharply with the more than 80% processing ratio in industrialized nations, indicating significant growth potential [19,20]. India processes around 2 to 2.5 million tons of fresh potatoes annually for chip production, with the majority of producers being small to medium-sized semi-automated operations. Only three to four large-scale, fully automated chip-manufacturing companies exist. French fries are an emerging product in India, and demand has grown rapidly due to the expansion of fast-food chains such as McDonald’s, KFC, and Pizza Hut [21]. In 2006, Canadian company McCain Foods established a large French fry-processing plant in Gujarat with an annual capacity of 30,000 tons, capturing 80% of the Indian frozen French fry market [22]. Other companies, such as Iscon Balaji Foods and Hyfun Foods, have also entered the frozen French fry market.

Dehydrated potato flakes in India are primarily used for processing “aloo bhujia” snacks. Iscon Balaji Foods Pvt. Ltd., Gujarat is the largest manufacturer, with an annual production capacity of 20,000 tons, followed by Vegit (a subsidiary of Melino Group) in Uttar Pradesh, producing 8000 tons per year. The industry is expanding its capacity to meet growing market demand [21].

### 4.4. Germany

Germany is one of the original countries of potato cultivation, with potatoes being grown since the 16th century [23]. In agriculture, Germany has achieved high levels of mechanization, becoming one of the world’s leaders in agricultural machinery. The country is home to many globally renowned agricultural machinery companies, such as CLAAS, Fendt, and KRONE.

Among the ten principal potato varieties in Germany, four are utilized for starch production, two for fresh consumption, and four for processing [23]. In the NWEC-05 region, which consists of Germany, France, the Netherlands, the United Kingdom, and Belgium, Germany occupies a dominant position in starch potato production. It contributes 43.3% to the total production in this region. Regarding consumption potato production, Germany’s share stands at 25.7%. This figure is remarkably similar to that of France and is the highest within the NWEC-05 [24].

Processed potato products like potato chips, mashed potatoes, potato cakes, and potato salads are extremely popular and come in a wide array of types. The per capita annual consumption, when converted to fresh potato equivalents, amounts to 56 kg. Potato processing constitutes around 70–80% of the total potato production in Germany, demonstrating a high level of industrialization [25]. Besides common processed products, Germany also manufactures various advanced products such as modified starches, boasting a technology level superior to that of China. Nevertheless, due to the high costs of mechanization, the number of low-yield farms is on the decline, which might pose an impact on the further development of the processing industry.

### 4.5. Poland

**Figure 3:**
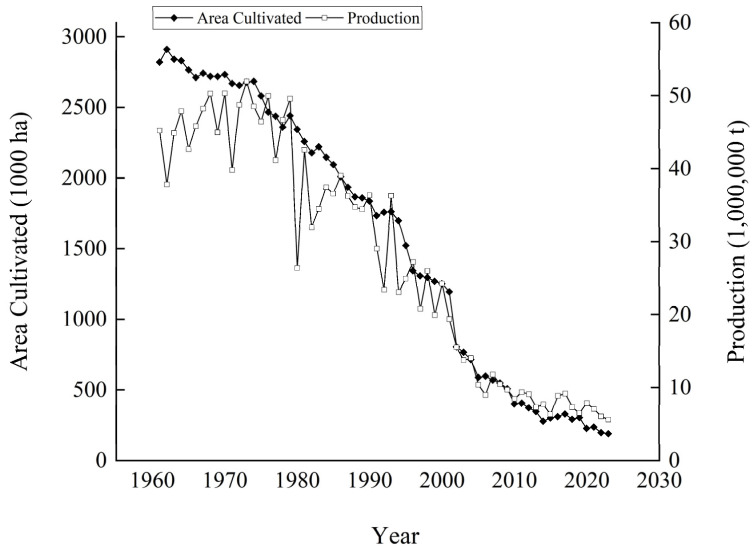
Overview of potato production in Poland from 1961 to 2023.

Poland is a traditional potato-producing powerhouse that once ranked second globally in terms of potato acreage and production [26]. According to the FAOSTAT database, the potato-cultivated area in Poland was 2,819,200 hectares in 1961 [8]. It dropped to 1,934,097 hectares in 1987 and further declined to 803 378 hectares in 2002. In 2023, the potato-cultivated area in Poland was merely 188,580 hectares, with a total output of 5.59 million tons (Figure 3).

The specific reasons why the potato-planting area in Poland gradually decreased from nearly 3 million hectares in the 1960s to less than 200,000 hectares in 2023 remain unknown, as there is a severe shortage of accessible literature and data. What exactly the potato industry in Poland has experienced is well worth studying. Perhaps it is because in the international market, potatoes from European countries such as Ukraine, the Netherlands, Germany, and France have advantages in terms of quality and price, putting pressure on Polish potatoes in the competition for international market share.

### 4.6. The Netherlands

Potato production in the Netherlands is geographically diverse, spanning across various regions of the country [23]. Five primary regions account for 70% of the total potato-cropped area. Drenthe, with 28,600 hectares, and Groningen, covering 27,000 hectares, are located in the northern part of the Netherlands, adjacent to the Lower Saxony region in Germany. These two regions mainly focus on starch potato production. North Brabant, in the southern Netherlands, encompasses 21,200 hectares; Zeeland, in the western part, has 19,000 hectares; and Flevoland, in the central Netherlands, covers 18,700 hectares [27].

In the Netherlands, approximately 27% of the potato crop is earmarked for starch production. This proportion is quite similar to that of Germany, where around 20% of the potato yield is dedicated to starch production. Collectively, these two countries account for nearly 80% of the total starch potato-cropped area in the NWEC-05 region.

When it comes to consumption potato production, the Netherlands holds the lowest share within the NWEC-05 region. Roughly 50% of the arable land designated for potato cultivation in the Netherlands is used for growing consumption potatoes. As a result, the Netherlands has the lowest proportion in both the production volume and the cropped area of consumption potatoes within the NWEC-05 region [28]. This distinct pattern of potato production in the Netherlands, with a relatively high proportion for starch production and a low share for consumption potato production, not only reflects the country’s agricultural production focus but also has a significant impact on its position in the regional potato-related market.

### 4.7. France

JeanPierre et al. [23] reported that 77.3% of the potato-cultivated area in France is used for the production of consumption potatoes. Among them, a third is for the domestic fresh market, a third is for the food-processing industry, and the remaining third is for export. Starch potatoes and seed productions each account for a certain proportion (11%). In terms of varieties, apart from Spunta for export, Fontane, Innovator, Challenger, Markies, and Bintje are commonly used in the food-processing industry. Starch production relies on Amyla and Kaptah Vandel. The fresh market is mainly dominated by Agata and Monalisa, and there are also many fresh food varieties selected for their good appearance after cleaning. Since 2005, many commercial potatoes in northern France have been specially supplied to Belgian French fry-processing plants, highlighting the influence of the Belgian potato industry on France.

### 4.8. United Kingdom

Potatoes are one of the most important economic crops in British agriculture [29]. They serve not only as a key food source but also as essential raw materials for the food-processing industry, particularly for products like French fries, potato chips, frozen foods, and other processed potato items [30]. According to the statistics of the FAO (https://www.fao.org/), the potato production and cultivated area in the United Kingdom are 4.7 million tons and 115,076 hectares, respectively, in 2023 [8]. Two major regions account for 46% of the potato cultivated area in the UK: East of England and Scotland, and these two regions produce most of the seed potatoes in the UK. Another 35% of the potato-cultivated area is composed of three regions: Yorkshire and the Humber, East Midlands, and West Midlands. The North West region and the South West region together account for approximately 11%.

The potato chip market in the UK is also expanding steadily. According to Mintel, the total value of the UK potato chip market is approximately GBP 1 billion, and this volume is expected to grow further by 2025 [31,32]. However, as global demand for potatoes and processed potato products increases, UK potato-processing companies are facing growing competition from producers in countries like the Netherlands and Belgium.

### 4.9. Belgium

**Figure 4:**
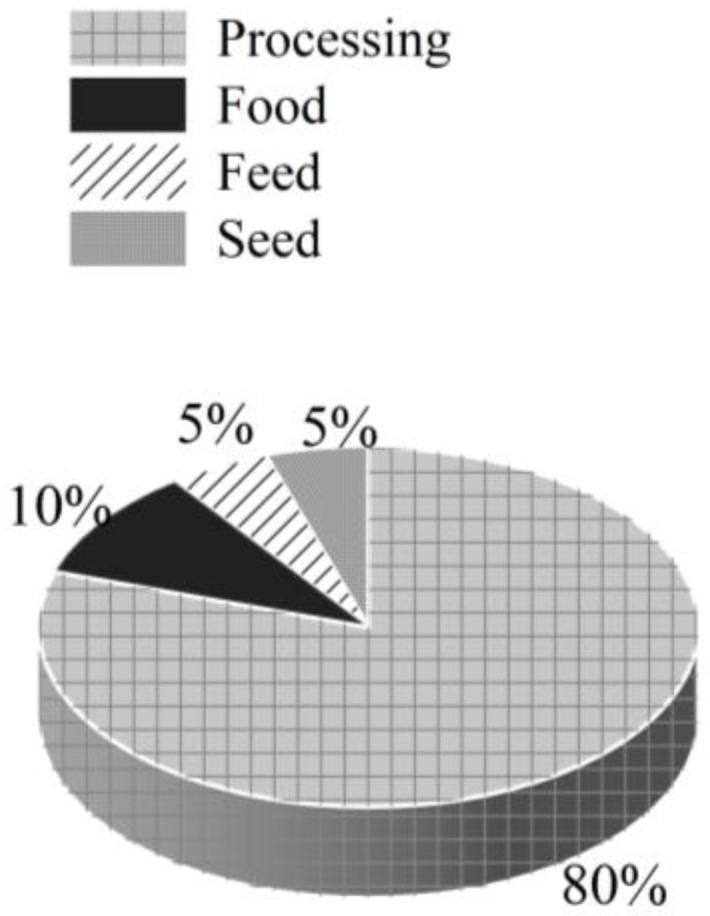
Distribution of potato utilization in Belgian.

Potato production in Belgium is primarily concentrated in the western regions of the country [23]. A large part of the potato production is located in the western part of the country close to the Nord-Pas-de Calais area in France. Two main regions represent 50% of the Belgian potato-cropped area: West Flanders in the Flemish region and Hainaut in the Walloon region [21]. Belgian potatoes are used for four main purposes: seed, feed, food, and processing. Approximately 80% of potatoes in Belgium are processed, 10% are consumed fresh, 5% are used for feed, and another 5% are allocated to seed production (Figure 4).

### 4.10. South Africa

**Figure 5:**
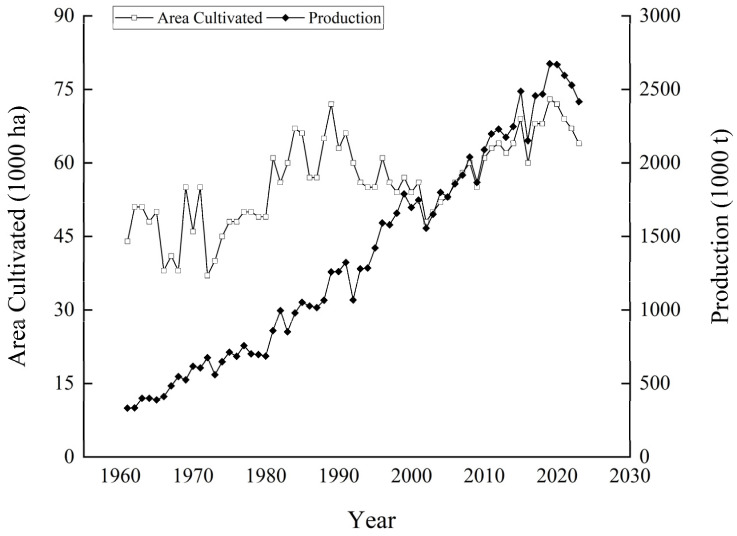
Overview of potato production in South Africa from 1961 to 2023.

Potatoes are the largest vegetable crop in South Africa [33]. According to data from the Food and Agriculture Organization (FAO) (https://www.fao.org/), South Africa’s potato production has shown steady growth over the past half century, increasing from 332,000 tons in 1961 to 2.42 million tons in 2023, representing a 627.60% relative increase. Specifically, the potato-cultivated area in 1961 was 43,682 hectares, while in 2023, it expanded to 63,510 hectares (Figure 5). This growth in cultivated area over the years reflects the dynamic development of the potato-growing industry in the region. Evidently, the substantial growth in South Africa’s total potato production is mainly attributed to the increase in yield. This could be the result of various factors such as the application of advanced agricultural technologies, improved potato varieties, and more scientific cultivation management methods. These elements have jointly promoted the improvement of potato production efficiency in South Africa, leading to a significant increase in the overall potato output despite the relatively stable cultivated area.

### 4.11. China

**I. Production factors**

According to FAO data, the global total potato production in 2023 was approximately 383 million tons, with China (93.4918 million tons) and India (60.142 million tons) ranking as the top two producers, respectively. China’s potato production areas cover regions such as Northeast, Northwest, and Southwest, forming core production areas like Ulanqab in Inner Mongolia and Dingxi in Gansu.

Accurate data on the production of the four main potato-processing categories in China are difficult to compile. According to the Potato Food Professional Committee of the China Food Industry Association, in 2023: frozen French fries: 1.274 million tons produced and sold; potato flakes (38 enterprises): production capacity of 340,300 tons; fresh-cut potato chips: production of 449,000 tons; potato starch: production of 565,200 tons (according to the Potato Starch Subcommittee of the China Starch Industry Association). Using material balance calculations, the output-to-input ratio for processing products is approximately: French fries: 2–2.5:1; fresh-cut potato chips: 4–5:1; potato flakes: 5–6:1; potato starch: 7–8:1. Based on these ratios, in 2023, China’s frozen French fries, fresh-cut chips, potato flakes, and potato starch consumed approximately 2.547 million, 1.702 million, 2.245 million, and 3.956 million tons of raw potatoes, respectively—a total of 10.45 million tons, accounting for 10.93% of the country’s total potato production in 2023.

**Gap with processing industry powerhouses.**

The processing industry level of potatoes in China is relatively low, with few types of processed products, most of which are primary products. There is a lack of high value-added products, and the development of deep-processed products faces technical challenges. On the one hand, the deep processing capacity of potatoes lags behind. In the processing of potato starch, the obtained dry matter contains 65% starch, 6.5% protein powder, 11% starch slurry water and 13% potato residue. On the other hand, China’s processing industry started relatively late. The innovation capacity in potato processing is weak. The technological innovation level and process level of processing enterprises are not high. They still remain at the processing methods of starch, whole flour, potato chips and French fries, etc. The product forms are relatively single, and the profit and product competitiveness are low [34].

**II. Demand conditions**

In recent years, China’s potato-processing industry has developed rapidly, with production areas all over the country. Processed products include starch, French fries, crisps, potato flakes and so on [35,36]. Currently, the market size of the domestic potato-processing industry is about RMB 38 billion. Between 2010 and 2022, edible consumption increased from 55.91 million tonnes to 69.1 million tonnes, a growth of 23.6%, processed consumption increased from 3.6 million tonnes to 4.86 million tonnes, a growth of 35.1%, and feed consumption decreased by 23.3%. Driven by the staple food strategy, the market penetration of potato buns, noodles, and other innovative products increased, and the production and sales of frozen French fries in 2023 reached 1.274 million tonnes. High-end product demand growth rate is significant; organic potatoes, low GI functional food, and other market segments average annual growth rate of more than 15%. However, the processing ratio (about 15%) is still far below the level of 50% in developed countries, indicating a huge market potential.

**Figure 6:**
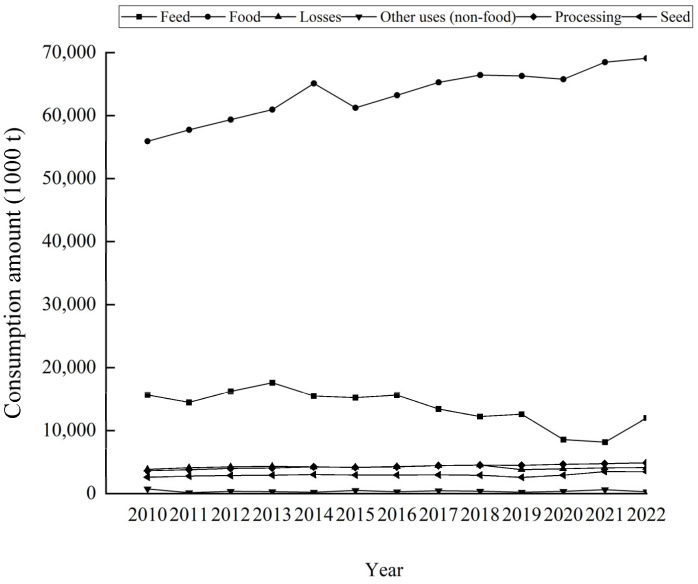
Potato consumption structure in China from 2010–2022.

Changes in the consumption structure not only reflect changing patterns of demand for agricultural products in China, but also have implications for the development of potato-related industries, such as the food-processing and seed production sectors. According to FAO data (Figure 6), the structure of potato consumption in China changed significantly between 2010 and 2022: feed consumption declined from 15.643 million tonnes in 2010 to 12.002 million tonnes in 2022, while food consumption increased from 55.911 million tonnes in 2010 to 69.109 million tonnes in 2022. The consumption of processed products increased from 3.661 million tonnes in 2010 to 4.865 million tonnes in 2022, and seed consumption increased from 2.584 million tonnes in 2010 to 3.432 million tonnes in 2022. While feed consumption and losses have declined, consumption for all other uses has increased during this period [37]. It is worth noting that the FAO data on China’s consumption structure for the period 2010–2022 are estimates, and changes in statistical methods have led to an underestimation of processed consumption. For example, FAO data show that in 2022, processing will account for only 5.09% of total potato production in China (95.57 million tonnes). However, according to FAO’s original statistical methodology, processing consumption in 2013 was 8.45 million tonnes, or 10.04 per cent of the total production of 84.21 million tonnes in that year.

**Gap with processing industry powerhouses.**

There is still much room for improvement in the proportion of potato processing and the market size in China. At present, China’s potato-processing capacity is insufficient, and there is a shortage of potato-processing varieties, especially those processed with whole flour [38]. The processing volume accounts for only about 10%, which is a huge gap compared with the processing proportions of the United States and the European Union [39]. Overall, the processing level of potatoes in China is still relatively backward. Moreover, the consumption mode of potatoes is mainly fresh consumption, accounting for more than 60% of the total consumption mode [34]. According to the data and information from the Food and Agriculture Organization of the United Nations (FAO), the actual consumption of potatoes in China has shown an upward trend in the past 50 years, but it is still lower than the actual consumption level of potatoes in many developed countries.

**III. Related and supportive industries**

China listed potato as the fourth largest staple crop in 2015, which helped promote the development of the potato-processing industry, leading to the establishment of a number of innovative processing enterprises. New products include dehydrated raw potato flour, potato bread, potato noodles, and potato buns [40,41,42,43,44]. Recently, the country’s first potato-pulp steamed bread production line was test-produced in Ulanchab, Inner Mongolia, and a potato rice production line was test-produced in Zhaotong, Yunnan, with important milestones achieved [45,46].

According to the China starch industry association potato-starch branch (https://www.siacn.org.cn/spdzi/B7ACVu4WjN.html, accessed on 5 November 2024) statistics, in 2023, China’s potato starch production was 565,200 tons.

Considering that many small-scale processing enterprises, new facilities, and expanded production capacity are not included in the statistics, together with other processed products (e.g., frozen hash browns, potato rice, and local specialties such as ‘potato fruits’ and ‘crisps’), the actual amount of potato processed is even higher [47]. For example, Lamb Weston (Inner Mongolia) officially started production of a new 10,800 tonne potato-cake line in 2023. Taking these factors into account, it is estimated that processed potatoes currently account for about 15 per cent of China’s total potato production.

**Gap with processing industry powerhouses**

The potato storage system abroad has become increasingly complete. Most potato-processing enterprises are equipped with dedicated storage warehouses to ensure that the loss rate is below 5%. During the storage and preservation of potatoes in China, due to factors such as storage facilities, management techniques, and preservation techniques, losses such as rot, sprouting, water loss, and low-temperature damage occur. The storage loss can be as high as 15%, and the storage and preservation technology of potatoes urgently needs to be improved. The preservation and storage of fresh sweet potatoes in China are relatively underdeveloped. Farmers in the northern regions mostly use cellars for storage, while those in the southern regions mainly continue to use indoor or underground stacking methods, which makes it difficult to meet the requirements of modern production. Only a small number of seed potatoes in China are stored in mechanical refrigeration constant-temperature warehouses, but the overall intelligence level of the facilities is relatively low [34].

**IV. Enterprise strategy, structure and peer competition**

The industry presents a “pyramid” competitive structure. Leading enterprises are accelerating their global layout. Although China has initially established a framework for potato brand building, its market influence and public recognition are still relatively low. Insufficient differentiation of brand characteristics, low brand aggregation, a lack of main products, and the non-standardization of market channels have led to an insignificant brand effect [48].

**Gap with processing industry powerhouses.**

On the one hand, the leading enterprises lack the driving force, and their capital and technological resources are limited, which leads to the limited operational scale of the leading potato enterprises and makes it impossible to effectively drive the modernization of the potato industry and increase the economic benefits of farmers. On the other hand, enterprises mainly focus on the production and sale of primary processed products, resulting in a relatively short industrial chain and narrow sales channels. In addition, China still needs to enhance the promotion of potato brands through new media. It has failed to fully utilize new media and online platforms to increase promotion efforts, and the communication methods of local brands have also failed to keep up with the new trend of networked life [34].

**V. Opportunities**

In 2014, the Ministry of Agriculture and Rural Affairs listed potatoes as the fourth major staple food crop for ensuring domestic food security after rice, wheat, and corn [49]. According to rural and agricultural big Data Network (http://www.agdata.cn/), more than 68 percent of China’s total potato production is used for fresh consumption and 8 percent for processing. China is rich in potato resources. By developing high economic value products such as French fries, potato chips, whole flour, and high-protein potato residue feed, and establishing a new regional potato production and utilization system in rural areas, it can effectively promote the improvement of the national potato value chain and the development of the industry.

**Gap with processing industry powerhouses.**

Relevant data show that in a certain year in the past, the per capita consumption of potatoes in China was approximately 41.2 kg, while during the same period, the per capita consumption of potatoes in some developed European countries reached 84.16 kg. From this perspective, there is still considerable room for growth in the domestic consumption of potatoes. It is estimated that the domestic consumption of potatoes will increase by 5% annually in the future [50].

**VI. The role of the government**

The first is institutional supply. The reward policy for major seed production counties was implemented. In 2021, Weiyuan County and others received special support. Thirteen regulations including the “Industry Standard for Potato Staple Food Products” were promulgated, and a full-process quality traceability system was established. The second is resource allocation. In the Inner Mongolia region, through the adjustment of the producer subsidy policy, potatoes have been included in the same subsidy scope as corn. The third is innovation incentives. Support joint research and development efforts by enterprises and research institutions, such as achieving a breakthrough in the potato and rice production line in Zhaotong, Yunnan Province in 2023.

**Gap with processing industry powerhouses**

The Dutch government has established a potato industry fund to support the protection of intellectual property rights of seed potatoes. Although potatoes are listed as staple food in China, the subsidy intensity is insufficient and the funds are used in a scattered manner. Domestic standards for potato processed products lag behind those of the European Union (such as the limit of acrylamide), resulting in obstacles to exports.

## 5. Comparison of Key Processed Products

### 5.1. French Fries

International market: North America and Europe are the primary production hubs for French fries globally [51]. In the international market, frozen French fries dominate due to several factors. The global fast-food industry’s continuous expansion, with the increasing number of international fast-food chain stores like McDonald’s and KFC, drives a stable and growing demand for frozen French fries. These fries are convenient for storage and transportation, meeting the large-scale and standardized ingredient supply requirements of fast-food enterprises. Moreover, countries like the United States and Canada have well-developed potato cultivation and processing industrial chains. Advanced cultivation techniques ensure a stable supply of high-quality raw materials, and advanced processing technologies guarantee product quality and production efficiency. Large-scale French fries production enterprises include McCain Foods Limited, Lamb Weston (including JVs and subsidiaries), J. R. Simplot, Aviko, and Farm Frites International [52]. McCain Foods is the world’s largest manufacturer of frozen potato products, with an estimated annual output of over 1 million tons, including a large quantity of French fries. McCain has more than 50 production bases globally and an annual sales volume of nearly USD 9 billion. Lamb Weston is the world’s second-largest producer of frozen potato products. According to Lamb Weston’s annual report, its global annual output is approximately 500,000 tons, most of which are frozen fries. Lamb Weston has multiple production plants and a distribution network worldwide. The annual output of J. R. Simplot is conservatively estimated to be over 350,000 tons. As one of the largest frozen French fries producers in the United States, Simplot’s frozen potato products have a wide influence in the North American and international markets. Aviko is one of the largest manufacturers of frozen potato products in Europe. According to its latest report, Aviko’s annual output is about 350,000 tons, most of which are frozen French fries and other potato products. Farm Frites produces approximately 300,000 tons of frozen potato products annually, including French fries. Farm Frites has a strong market share in the European and Middle-East markets and has also expanded to Asia and other regions.

As environmental and sustainability concerns rise, French fry producers worldwide have increasingly prioritized green packaging solutions to reduce carbon emissions and resource waste during production [53].

Chinese market: In China, French fries are primarily categorized into frozen French fries and packaged snack fries, with frozen French fries dominating the market. The dominance of frozen French fries in the Chinese market can be attributed to the booming domestic fast-food industry. The rise in local fast-food brands and the increasing demand for fast-food delivery services have led to a surge in the demand for frozen French fries, which are an essential ingredient in fast food. Additionally, the abundant supply of potatoes in China provides a stable and cost-effective raw material source for frozen French fry production. Moreover, consumers’ growing acceptance of convenient foods and the simplicity of preparing frozen French fries at home have further propelled their market dominance. Production has grown rapidly due to rising domestic demand and the expansion of fast-food chains [36]. With a sufficient supply of potatoes, China provides an ample raw material base for the development of the French fry industry. The rapid growth of the food delivery sector, particularly among younger consumers, continues to drive demand, making French fries an essential part of daily life as a staple in fast-food establishments [54].

**Figure 7:**
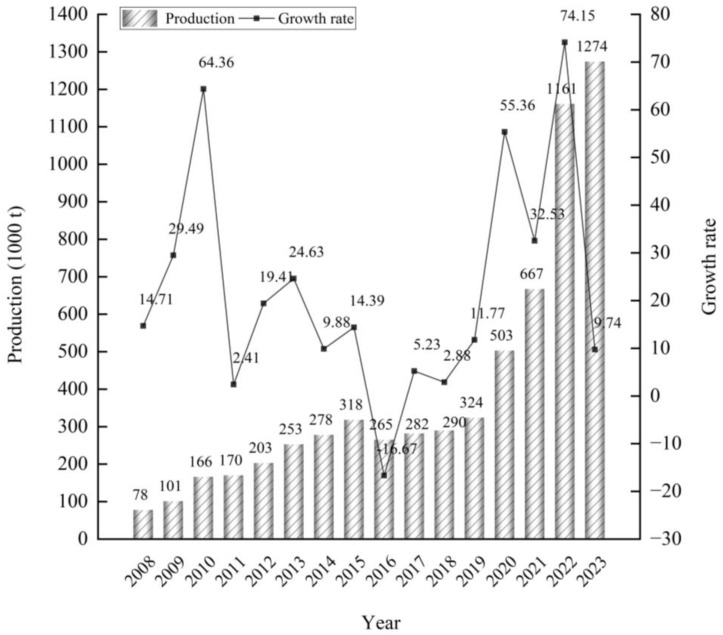
The production capacity of frozen French fries of the eight major enterprises in China from 2008 to 2023.

According to the Potato Food Professional Committee of the China National Food Industry Association (http://www.cnfia.cn/), in 2023, China’s eight major French fry manufacturers produced and sold 1.2737 million tons of French fries. These manufacturers include Beijing Kaida Hengye Agricultural Technology Development Co., Ltd. (Beijing, China) (500,000 tons), SnowValley Food Hebei Co., Ltd. (Zhangjiakou, China) (280,000 tons), McCain Foods (Harbin) Co., Ltd. (Haerbin, China) (175,000 tons), Lamb Weston Potatoes (Inner Mongolia) Co., Ltd. (Inner Mongolia, China) (160,000 tons), Landun Xumei Food Co., Ltd. (Taiyuan, China) (61,700 tons), Aviko (Inner Mongolia) Food Co., Ltd. (Inner Mongolia, China) (50,000 tons), Simplot (China) Foods Co., Ltd. (Yinchuan, China) (44,000 tons), and Inner Mongolia Mengshu Food Technology Co., Ltd. (Inner Mongolia, China) (3000 tons) (Figure 7). Additionally, Snowvalley Liupanshan Food (Ningxia) Co., Ltd., (Guyuan, China) established in 2021, has commenced operations with a production capacity of 400,000 tons.

The French fry industry in China started relatively late but has experienced rapid market growth. However, much of the equipment and processing technology relies on imports, such as fully automated potato strip cutting and drying systems from the Netherlands, which dominate the Chinese market. Recently, domestic companies like SnowValley Agriculture (Zhangjiakou, China) and Inner Mongolia Kaida Hengye Food Co., Ltd. (Inner Mongolia, China) have introduced unmanned production lines. While Chinese companies have made notable progress in French fry production, there is still room for improvement in product quality and production efficiency.

### 5.2. Potato Chips

In the international market, traditional fried potato chips dominate mainly because of consumers’ long-standing taste and texture preferences. Over time, a strong consumption habit has formed around these chips. Technologically, the production technology for fried potato chips is mature and cost effective, enabling large-scale production to meet market demands. Additionally, well-known international brands like Lay’s and Pringles have established a strong market presence through extensive marketing and advertising campaigns, reinforcing their products’ dominance. From the 1920s onwards, a series of technical innovations have continuously promoted the development of the potato chip manufacturing industry to its current advanced level. In the 1920s, the mechanical potato peeler was invented, and potato chips started to be packaged in sealed bags. In the 1950s, seasoning technology emerged, adding various flavors to potato chips. In 1985, microprocessor-controlled weighing heads were introduced, which improved the accuracy of product weighing during production. In 1990, optical sorting technology was used to remove defective products, ensuring product quality, and in 1995, nitrogen-fill technology was adopted to preserve the freshness of potato chips for a longer time. Lay’s and Pringles, which are made from fresh potatoes and potato flour, are packaged in cans. These technological advancements have not only enhanced the production efficiency but also improved the quality and taste of potato chips, making them a popular snack worldwide. As health awareness among consumers continues to grow, there is increasing demand for low-fat, low-salt, minimally processed, and functional potato chips [55].

Chinese market: The Chinese potato chip market includes various types such as fresh-cut chips, composite fried chips, composite baked chips, hard and crunchy chips, biscuit chips, and puffed chips, with a total annual output of approximately 2 million tons. The primary consumer group consists of young people, with demand concentrated in economically developed regions. Despite the rise in domestic brands, the high-end market is still dominated by international players such as Lay’s and Pringles. Other competing brands include Three Squirrels, Oishi, Copico, Shuyuan, Bugles, Pringles, Haoyouqu, Orion, Calbee, QinQin, Cadina, Xiao Wangzi, I’believe, Zidi, Royce, and others [56]. While there is a diverse range of potato chip products, emerging healthy potato chips, such as baked chips and low-oil, low-salt chips, are gradually gaining popularity. This is mainly due to the increasing health awareness among Chinese consumers, who are more inclined to choose low-fat and low-salt food options. Domestic enterprises have responded to this trend by investing in research and development to produce healthy potato chips. For example, some brands adopt vacuum low-temperature frying technology, which not only reduces the fat content but also the acrylamide content, making the products healthier. These brands also leverage online and offline sales channels to expand their market reach, thus increasing their market share.

In recent years, driven by increasing demand for healthier food options, domestic companies have started adopting low-fat, low-salt production methods. Some have also developed baked and additive-free chips. The variety of potato chips is expanding, with innovative flavors and gluten-free options being introduced to cater to niche consumer groups. Among domestic brands, Copico occupies a significant share of the snack market, while Xiao Wangzi has improved chip taste and quality through its proprietary secondary spiral extrusion pre-gelatinization technology. Additionally, brands like Qinqin Potato Chips from Fujian Qinqin Holdings Co., Ltd. (Jinjiang, China) and Cuishengsheng from Harmony Foods (Tianjin) Co., Ltd. (Tianjin, China) are intensifying competition. Cuishengsheng adopts vacuum low-temperature frying technology, resulting in lower fat content and acrylamide content, making it healthier [57,58]. It is exported to more than 30 countries and regions, such as Japan, South Korea, Singapore, Thailand, Malaysia, and the Philippines.

According to the Potato Food Professional Committee of the China National Food Industry Association (http://www.cnfia.cn/), in 2023, the output of fresh-cut chips in China reached 449,000 tons, with a sales revenue of 9.16 billion yuan. Composite fried chips reached 419,000 tons, with sales of 8.38 billion yuan. By 2024, the output of fresh-cut chips is expected to reach 714,000 tons, with a projected revenue of 14.58 billion yuan, while composite fried chips are forecasted to reach 453,000 tons, generating 9.36 billion yuan in revenue.

### 5.3. Flakes, Granules, and Pellets of Potatoes

In the international market, potato flakes and related products dominate in certain food-industry segments. Their popularity is mainly due to their excellent storage and transportation properties, as well as good rehydration ability. They can be easily incorporated into a variety of food products, such as instant mashed potatoes and some baked goods, providing a convenient and stable ingredient source for food manufacturers. In the food-processing industry, their use can simplify production processes and improve production efficiency. For example, in the production of instant food, potato flakes can be quickly rehydrated to provide a potato-based component, meeting the needs of consumers for quick and easy meals. Notably, potato flakes played a crucial role during both World War I and World War II, particularly in the latter, when they were widely used as military rations [59].

During World War I, severe food shortages and logistical challenges prompted many countries to seek more efficient methods of food preservation and transportation. As a lightweight, easily stored, and long-lasting dehydrated food, potato flakes emerged as an ideal solution. Their significance grew even further during World War II, as wartime supply chains became more strained. Potato flakes were not only a staple in military combat rations, providing soldiers with a convenient and durable food source, but also served as a high-nutrition alternative for civilians facing food scarcity. By processing potatoes into flakes, their bulk was significantly reduced, facilitating large-scale transportation and storage.

Beyond military use, potato flakes were also incorporated into the production of convenience foods for both war-torn regions and civilian populations in the rear. Additionally, the war years saw significant advancements in potato flake manufacturing technology, leading to improved production efficiency and product quality. European countries, in particular, made notable strides in this field, establishing themselves as global leaders in dehydrated potato product processing [60].

**Figure 8:**
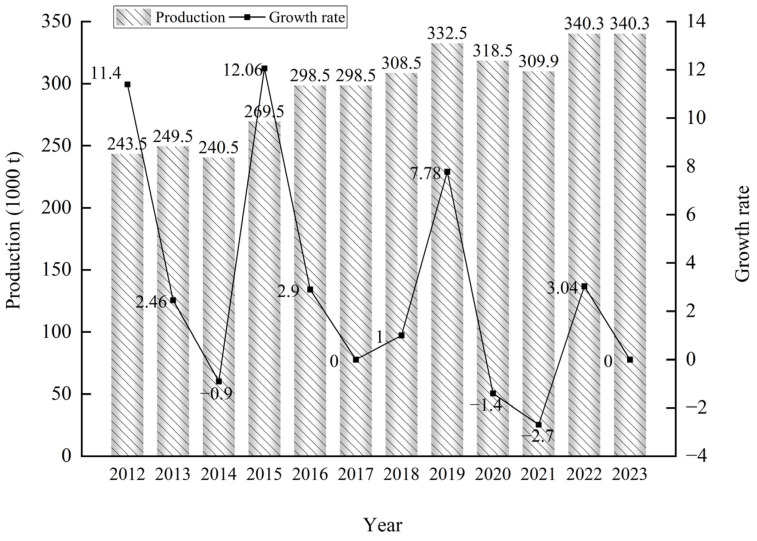
Production capacity of potato flakes of 38 major enterprises in China from 2012 to 2023.

Chinese market: In China, potato flake production is mainly concentrated in the northeastern and northwestern regions. For instance, companies in Gansu and Xinjiang benefit from local rich potato resources and advanced production lines. Their products have a high market share in the domestic food-processing market because they can meet the strict requirements of food enterprises in terms of product quality, price, and supply stability. As the domestic food industry continues to grow, the demand for potato flakes is increasing, and these leading enterprises are well-positioned to meet this demand through their scale and quality advantages. With the expansion of potato cultivation areas, several enterprises have begun upgrading and transforming their operations. According to the Potato Food Professional Committee of China National Food Industry Association (http://www.cnfia.cn/), in 2023, the total production capacity of 38 major potato flake manufacturers in China reached 340,300 tons (Figure 8). Major producers include Gansu Aviko Potato Processing Co., Ltd. (Zhangye, China)(30,000 tons), Gansu Dali Food Co., Ltd. (Wuwei, China)(21,000 tons), Xinjiang Da Luosu Food Co., Ltd. (Beitun, China)(20,000 tons), Zhangjiakou Yanbei Potato Industry Development Co., Ltd. (Zhanjiakou, China)(20,000 tons), Hongji Agriculture Co., Ltd., Zhangjiakou, Hebei (Zhangjiakou, China)(20,000 tons), and Keshan County Fumin Agricultural Industry Development Co., Ltd. (Heilongjiang, China)(20,000 tons). In October 2024, Snowvalley Liupanshan Food (Ningxia) Co., Ltd. (Guyuan, China) completed testing and began production of a new potato flake production line with an annual capacity of 25,000 tons.

Additionally, several new potato flake production lines are planned for 2024, including a 15,000 ton capacity line by Gansu Dali Food Co., Ltd. (Wuwei, China), a 10,000 ton line by SnowValley Food Co., Ltd. (Zhangjiakou, China), and another 10,000 ton line by Kaida Hengye (Inner Mongolia, China), all of which are expected to be operational by 2025.

### 5.4. Starch

International market: In 1820, the production of potato starch began in Hillsborough County, New Hampshire, USA [61]. Currently, global potato starch production is mainly concentrated in Europe, particularly in Germany and the Netherlands [23]. In the food industry, its unique physical and chemical properties, such as high gelatinization degree and excellent thickening and stabilizing capabilities, make it an ideal choice for high-end food production, like premium baked goods, sauces, and meat products. In industrial applications, potato starch is widely used in papermaking and textile industries. It can improve the strength and gloss of paper and enhance the texture and dyeing properties of textiles. German and Dutch potato starch enterprises, with their advanced processing technologies, can produce high-quality potato starch products that meet the stringent requirements of these high-end markets and industrial applications, thus dominating in relevant fields. In countries such as those in Europe, the United States, and Japan, the production of modified starch and deeply processed potato starch products includes thousands of varieties. These nations utilize equipment and processing technologies that enable efficient, low-energy production with minimal environmental impact, significantly improving production efficiency and product quality. Notable companies in this field include Emsland-Stärker in Germany, AVEBE in the Netherlands, and KMC in Denmark. However, global climate change and resource constraints may affect potato cultivation and supply, particularly in Europe and the United States [62].

**Figure 9:**
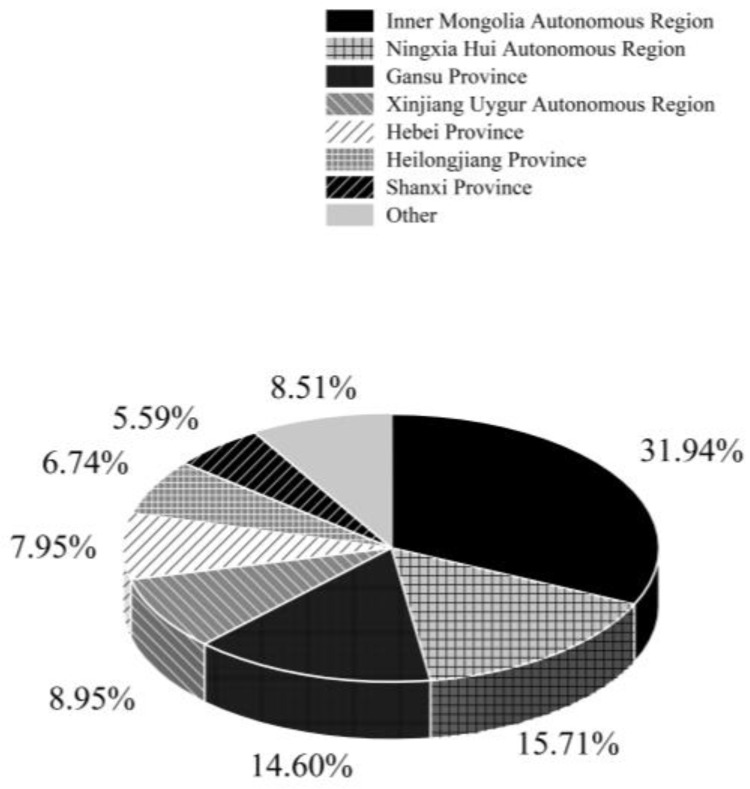
Proportion of potato starch production by provinces in 2023.

Chinese market: China’s annual production capacity for potato starch is approximately 2 million tons, primarily used in the food industry, pharmaceuticals, and papermaking. In the food industry, its natural and additive-free characteristics meet consumers’ pursuit of healthy food. It is widely used in the production of some high-end and specialty foods, such as certain types of premium vermicelli and pastries. In the pharmaceutical field, its good stability and safety make it an important excipient in some medications. In papermaking, with the increasing emphasis on environmental protection, potato starch, as a biodegradable natural raw material, has broad application prospects. Enterprises in Gansu, Ningxia, and other regions have capitalized on local potato resources and technological innovation to improve product quality, meeting the diverse needs of these industries and gaining a competitive edge in the domestic market”. According to the Potato Starch Branch of the China Starch Industry Association (https://www.siacn.org.cn/spdzi/B7ACVu4WjN.html, accessed on 5 December 2024), potato starch production in China ranged between 450,000 and 700,000 tons from 2017 to 2022. In 2023, production reached 565,200 tons, representing a 16% year-on-year increase. Overall, China’s potato starch production has shown a steady upward trend. Potato starch processing in China is mainly concentrated in the northwest, northeast, and north regions, with Gansu, Ningxia, Xinjiang, and Inner Mongolia Autonomous Region being the leading production areas (Figure 9). In 2023, these four provinces produced a combined total of 402,500 tons of potato starch, accounting for 82.44% of the national total.

Due to the regional distribution of potato cultivation, potato starch enterprises in China are also geographically dispersed [63]. Most companies operate on a relatively small scale, with the highest concentration of enterprises located in the main production provinces. Outside these areas, companies are scattered across other provinces.

## 6. Key Characteristic Factors of Major High—Value—Added Potato Processing Countries

### 6.1. R&D Investments

Some countries have consistently invested in research and development (R&D) to innovate and optimize potato-processing technologies. For example, Belgium works on enhancing product quality, variety, and nutritional value, especially in potato chips and fries [64]. Canada invests in developing unique potato varieties for specific processing needs and researching sustainable farming practices. Germany is a leader in starch-related R&D, creating advanced modified starches for diverse industrial uses [23].

### 6.2. Infrastructure

The United States has advanced irrigation systems for stable potato yields and high-tech processing facilities, along with an extensive transportation network. Belgium’s specialized food-processing infrastructure features advanced machinery for precise processing and well-maintained storage facilities. Canada’s robust agricultural infrastructure includes cold storage for continuous raw material supply and modern processing equipment. Germany’s highly mechanized agricultural infrastructure enables high-yield potato production and large-scale, precise processing in the industry.

### 6.3. Supply-Chain Logistics

In the United States, growers have long-term contracts with processors, and advanced inventory management systems optimize storage and transportation. Belgium’s strategic location and well-connected transportation networks, along with a strong supplier–distributor network, ensure a smooth supply chain. Canada’s processors have close relationships with growers, and its logistics focus on maintaining the cold chain for frozen products. Germany’s well-developed transportation network and strict quality-control measures throughout the supply chain contribute to its success.

### 6.4. Policy Incentives

The U.S. government offers subsidies to farmers and tax breaks to processing companies [65]. Belgium provides grants for SMEs to invest in R&D and promotes international trade. Canada has agricultural support programs and export–promotion policies. Germany encourages sustainable farming and funds innovation projects in the potato-processing industry. These policies create a favorable environment for industry growth and competitiveness.

## 7. Quality Management and Automatic Control

### 7.1. Quality Control

Quality management is of importance in potato processing to ensure product consistency, safety, and fit with regulatory standards. In order to improve product quality and meet consumer expectation, the Hazard Analysis Critical Control Point (HACCP) system in a large-scale potato processing was suggested to apply to reduce microbial contamination [66]. Additionally, advanced techniques such as near-infrared spectroscopy was established as an effective tool to obtain prediction equations of these potato quality parameters [67]. Furthermore, a relatively scientific and complete food quality and safety management system applicable to clean potato-processing enterprises by integrating ISO 22000 standards is also required [68]. This system ensures the safe and reliable supply of food, providing a systematic management model for food safety. At the same time, it can maintain the freshness and nutritional value of potatoes to the greatest extent, improve the appearance quality of products, extend the shelf life, and meet the demand for potatoes of urban residents.

### 7.2. Automation Level

Automatic control systems could make potato processing more efficient, precise, and available on a larger scale. For example, in the process of potato chip production, automated fryers equipped with real-time sensors and feedback loops can adjust temperature and frying time based on the moisture content and thickness of the slices, ensuring consistent product quality. A fully automated production line was designed by ElMasry et al. [69], which utilized machine-vision systems to detect and remove defective potatoes and ensured the classified well-shaped potatoes by size, achieving 100% accuracy. In the production of carbohydrate-rich food items such as potato chips and French fries, a compound known as acrylamide can be formed; therefore, a deep learning-based computer vision framework for the automatic detection of the presence of acrylamide was proposed by Maurya et al. [70]. These advancements underscore the transformative potential of automatic control systems in achieving sustainable and efficient potato-processing operations.

## 8. Sustainable Practices in the Global Potato-Processing Industry

### 8.1. Circular Economy

In some European countries like the Netherlands and Germany, potato-processing companies are finding ways to reuse and recycle by-products [71]. Potato peels and other waste materials from the processing of potato fries, chips, or starch are being converted into animal feed, biofuels, or biodegradable materials. This not only reduces waste sent to landfills but also creates additional value from that previously considered waste. Some companies have established closed-loop systems where the waste generated during potato processing is used as raw material for other industries, thus minimizing the overall environmental impact [72].

### 8.2. Green Packaging

In North America and Europe, major potato-processed food companies are shifting towards packaging solutions that are more environmentally friendly. For French fries and potato chips, companies are using biodegradable or compostable packaging materials [73]. For example, PepsiCo has introduced 100% compostable packaging for its snack products. These materials can break down naturally in the environment, reducing the amount of plastic waste. Additionally, some companies are also reducing the amount of packaging used per product, further minimizing their environmental footprint.

### 8.3. Waste Valorization

Waste valorization techniques are being employed to convert potato-processing waste into valuable products, including extracting starch, proteins, and fibers from waste streams for use in food, pharmaceuticals, or industrial applications, thereby reducing environmental impact and enhancing resource efficiency. For the potato peels produced in the processing, the Aviko has converted those into biogas and animal feed, thereby reducing landfill use. In 2015, China implemented strict environmental protection policies for potato starch factories, leading to the closure of many small and medium-sized potato starch factories [72]. Almost all large-scale potato starch factories adopted the wastewater treatment technology developed by the Lanzhou Institute of Chemical Physics, Chinese Academy of Sciences. Specifically, the thermal flocculation method is first used to recover protein from the separated juice water during potato starch processing. Then, the de-proteinized water is either returned to the fields as organic fertilizer water or discharged after meeting the standards through biochemical treatment.

As the global population is anticipated to reach 10 billion by 2050 and climate change has already started to pose challenges to our ability to generate enough food, the necessity for the sustainable transformation of agri-food systems has reached an all-time high [74]. As is reported by the International Potato Center (CIP), more than 300 million people below the poverty line from developing countries still rely on root, tuber, and banana crops, including sweet potato and potato. The market value of those consumed crops reaches USD 339 billion [75]. Therefore, CIP partnered with CGIAR centers for the “Roots, Tubers and Bananas (RTB)” program, which increased food production with improved varieties in multiple countries, like China, India, and Nepal. Over the last 10 years, there has been an increase of over 50% in potato production in seven Asian countries, including Bangladesh, China, India, Indonesia, Nepal, Pakistan, and Vietnam, and the value of farmgate sales has more than tripled, reaching approximately USD 37 billion annually [76]. However, in order to face the global challenges like climate change and crop pests and disease in future, the protection of genetic diversity using cryopreservation is necessary.

## 9. Conclusions

This report analyzes the differences between the potato-processing industries in China and other countries, and finds that there is still significant room for development in China’s potato-processing industry in terms of market scale, production equipment, technical management, and product innovation. In addition, compared with the Western countries where the potato-processing industry is well developed, in which the processing proportion of potatoes typically ranges from 60% to 80%, the processing proportion of potatoes in China is relatively low, standing at only about 15%. In China, the diet mainly consists of fresh vegetables, and the demand for potato-processed foods is relatively low. There are gaps in product quality, brand awareness, and marketing methods, and they face strong international competition pressure, which is not conducive to the expansion of the processing industry. In terms of adopting automated technologies, China should increase its efforts in introducing advanced intelligent production equipment. It should not only bring in mature automated production lines from abroad but also encourage domestic enterprises to independently develop equipment suitable for the local production environment, so as to improve production efficiency, reduce labor costs, and enhance the stability of product quality. Regarding the strengthening of new variety research and development, for potato chip processing, China can cultivate varieties with moderate starch content, good taste, and excellent color after frying. For starch production, it can develop varieties with high starch content and easy extraction. At the same time, it is necessary to strictly control product quality, follow international standards, and meet the needs of consumers in different countries and regions. Some high-quality Chinese French fries and potato chip brands can establish cooperative relationships with foreign distributors by participating in international food exhibitions and gradually open up the international market.

In conclusion, according to the analysis using Porter’s Competitive Diamond framework, China benefits from abundant natural resources such as vast arable land and a large domestic demand markets, but it faces some limitations in advanced agricultural technologies, efficient supply chains, and international competitiveness. To enhance the competitiveness of Chinese potato industry, China should focus on improving factor quality, fostering innovation in seed technology, enhancing the development of industries, and strengthening collaboration between farmers, processors, and retailers. Additionally, learning from leading potato-producing countries could provide valuable insights for policy formulation and industry development, thus contributing significantly to the global markets. With the changes in consumption concepts and technology, China’s potato-processing industry has good development opportunities. It should continuously enhance its industry competitiveness to achieve high-quality development of the potato-processing industry.

## Figures and Tables

**Table 1:** Comparison of key statistical data of major global potato-producing countries.

Comparison Items	China	United States	Canada	India	Germany	Poland	The Netherlands	France	United Kingdom	Belgium	South Africa
Potato production in 2023 (tones)	93,491,819	19,992,090	6,543,393	60,142,000	11,607,300	5,590,030	6,491,890	8,606,490	4,704,724	4,021,930	2,415,638
Potato harvest area in 2023 (hectares)	4,571,531	388,580	156,642	2,332,000	264,700	188,580	155,340	204,010	115,076	95,700	63,510
Processing proportion	15%	64.46%	68%	7%	70–80%	-	-	-	-	80%	-

**Table 2:** Comparison of potato and potato products trade (exports and imports) in major potato-producing countries in 2023 (metric tonne).

HS Code	China		United States		Canada		India		Germany		Poland		The Netherlands		France		United Kingdom		Belgium		South Africa	
	EXP	IMP	EXP	IMP	EXP	IMP	EXP	IMP	EXP	IMP	EXP	IMP	EXP	IMP	EXP	IMP	EXP	IMP	EXP	IMP	EXP	IMP
070190 ^a^	390,043	-	540,339	559,560	602,963	268,858	516,845	3199	2,173,624	463,573	44,512	147,210	1,654,477	1,806,734	2,625,411	489,392	165,535	-	812,299	-	136,814	3
110520 ^b^	358	11,284	85,638	48,542	9216	7818	28,648	1676	168,579	38,455	14,362	37,067	90,521	26,984	5544	34,207	3772	35,786	78,751	27,679	1456	464
110813 ^c^	4612	22,365	8497	140,369	21,885	12,247	4309	2667	263,886	63,864	79,863	22,100	188,479	120,221	-	34,850	1897	66,227	38,263	-	325	2883
200410 ^d^	134,399	39,502	886,605	1,459,410	1,351,163	49,176	116,077	1	318,658	384,734	206,166	167,473	1,975,457	350,697	447,134	643,484	-	828,017	2,851,622	126,874	8240	45,373
200520 ^e^	10,747	2440	107,682	78,412	48,796	44,048	1127	5	59,210	144,868	57,419	16,709	293,810	58,819	29,736	116,006	-	64,417	122,684	35,981	13,041	1236

^a^ Potatoes (other than seed), fresh or chilled; ^b^ Flakes, granules, and pellets of potatoes; ^c^ Potato starch; ^d^ Potatoes prepared or preserved other than by vinegar or aceic acid, frozen; ^e^ Potatoes prepared or preserved other than by vinegar or aceic acid, not frozen.

## Data Availability

No new data were created or analyzed in this study. Data sharing is not applicable to this article.

## References

[1] Buono V., Paradiso A., Serio F., Gonnella M., De Gara L., Santamaria P. (2009). Tuber quality and nutritional components of “early” potato subjected to chemical haulm desiccation. J. Food Compos. Anal..

[2] Chung I.M., Kim J.K., Jin Y.I., Oh Y.T., Prabakaran M., Youn K.J., Kim S.H. (2016). Discriminative study of a potato (*Solanum tuberosum* L.) cultivation region by measuring the stable isotope ratios of bio-elements. Food Chem..

[3] Zaheer K., Akhtar M.H. (2016). Potato production, usage, and nutrition—A review. Crit. Rev. Food Sci. Nutr..

[4] Granato D., Branco G.F., Nazzaro F., Cruz A.G., Faria J.A.F. (2010). Functional foods and nondairy probiotic food development: Trends, concepts, and products. Compr. Rev. Food Sci. Food Saf..

[5] Sinha R., Khot L.R., Schroeder B.K., Si Y.S. (2017). Rapid and non-destructive detection of *Pectobacterium carotovorum* causing soft rot in stored potatoes through volatile biomarkers sensing. Crop Prot..

[6] The Global Times. https://m.toutiao.com/article/7450630667597087283/?upstream_biz=doubao&show_loading=0&webview_progress_bar=1.

[7] Wang Z.J., Liu H., Zeng F.K., Yang Y.C., Xu D., Zhao Y.C., Liu X.F., Kaur L., Liu G., Singh J. (2023). Potato processing industry in China: Current scenario, future trends and global impact. Potato Res..

[8] FAOSTAT. https://www.fao.org/faostat/en/#data/QCL.

[9] Date Bridge. https://www.databridgemarketresearch.com/reports/global-potato-processing-market.

[10] Statista. https://www.statista.com.

[11] UN Comtrade Database. https://comtrade.un.org/.

[12] Pavlista A.D., Ojala J.C. (2023). Potatoes: Chip and French fry processing. Processing Vegetables.

[13] Kirkman M.A. (2007). Global markets for processed potato products. Potato Biology and Biotechnology.

[14] Barrett R., Robinson A., Thornton M., VanderZaag P. (2023). Potato production in the United States and Canada. Potato Production Worldwide.

[15] Arslan M., Xiaobo Z., Shi J., Rakha A., Hu X., Zareef M., Zhai X.D., Basheer S. (2018). Oil uptake by potato chips or French fries: A review. Eur. J. Lipid Sci. Technol..

[16] Wohleb C.H., Waters T.D., Crowder D.W. (2021). Decision support for potato growers using a pest monitoring network. Am. J. Potato Res..

[17] Corsini D.L., Brown C.R. (2001). Important potato cultivars. Virus and Virus-Like Diseases of Potatoes and Production of Seed-Potatoes.

[18] Keijbets M.J.H. (2008). Potato processing for the consumer: Developments and future challenges. Potato Res..

[19] Gupta V.K., Luthra S.K., Bhardwaj V. (2020). Potato processing varieties in India. Indian Hortic..

[20] Sahu P.K., Das M., Sarkar B., Adarsh V.S., Dey S., Narasimhaiah L., Mishra P., Tiwari R.K., Raghav Y.S. (2024). Potato production in India: A critical appraisal on sustainability, forecasting, price and export behaviour. Potato Res..

[21] Kaur S., Aggarwal P., Kaur N. (2024). Evolution of Indian frozen french fry industry: Industrial constraints, challenges and future prospects. Potato Res..

[22] The Times of India. https://timesofindia.indiatimes.com/city/ahmedabad/gujarat-drives-indias-french-fries-export/articleshow/73718505.cms.

[23] Goffart J.P., Haverkort A., Storey M., Haase N., Martin M., Lebrun P., Ryckmans D., Florins D., Demeulemeester K. (2022). Potato production in Northwestern Europe (Germany, France, the Netherlands, United Kingdom, Belgium): Characteristics, issues, challenges and opportunities. Potato Res..

[24] BSA. https://www.bundessortenamt.de/bsa/sorten/beschreibende-sortenlisten/download-bsl-im-pdf-format.

[25] Farooq K., Mubarik A., Aqsa Y. (2020). Potato cluster feasibility and transformation study. Cluster Development Based Agriculture Transformation Plan Vision-2025. Project.

[26] Mondy N., Lisinska G., Leszczynski W. (1990). Potato Science and Technology.

[27] Eurostat. https://ec.europa.eu/eurostat/databrowser/view/APRO_CPSH1__custom_713410/default/table?lang=en.

[28] CBS. https://www.cbs.nl/en-gb/figures/detail/7100eng.

[29] Adesina O.S., Thomas B. (2020). Potential impacts of climate change on Uk potato production. Int. J. Environ. Clim. Change.

[30] McGill C.R., Kurilich A.C., Davignon J. (2013). The role of potatoes and potato components in cardiometabolic health: A review. Ann. Med..

[31] Mintel. https://store.mintel.com/report/uk-crisps-savoury-snacks-and-nuts-market-report-2022.

[32] Tridge. https://www.tridge.com/intelligences/chips/GB.

[33] Hanekom J.W., Willemse B.J., Strydom D.B. Structure, conduct and performance in the South African potato processing industry. Proceedings of the 2010 AAAE Third Conference/AEASA 48th Conference.

[34] Wei M., Xu H., Zhang N., Si H., Tang X. (2024). Investigation on Current Situation of Potato Processing Industry and Relevant Countermeasure in China. Chin. Potato.

[35] Wang Q.B., Zhang W. (2010). An economic analysis of potato demand in China. Am. J. Potato Res..

[36] Yang Y.L., Guo Y.Z., Sun J.M. (2017). Present status and future prospect for potato industry in China. J. Agric. Sci. Technol..

[37] FAOSTAT. https://www.fao.org/faostat/en/#data/FBS.

[38] Wang T., Ma S., Jin G. (2022). Research Progress in Development of Potato Granule and Flake Processing Varieties in China. Chin. Potato.

[39] Wang S., Lv H., Lu T., He Y., Lv C., Yang B. (2022). The Current Development Status and Suggestions of China’s Potato Processing Industry. Agric. Eng..

[40] Zhang H., Xu F., Wu Y., Hu H.H., Dai X.F. (2017). Progress of potato staple food research and industry development in China. J. Integr. Agric..

[41] Liu X.L., Mu T.H., Sun H.N., Zhang M., Chen J.W. (2016). Influence of potato flour on dough rheological properties and quality of steamed bread. J. Integr. Agric..

[42] Jin C.Y., Xu D., Zeng F.K., Zhao Y.C., Yang Y.C., Gao G.Q., Wen G.H., Liu G. (2017). A simple method to prepare raw dehydrated potato flour by low-temperature vacuum drying. Int. J. Food Eng..

[43] Zeng F.K., Liu H., Yu H., Cheng J.C., Gao G.Q., Shang Y., Liu G. (2019). Effect of potato flour on the rheological properties of dough and the volatile aroma components of bread. Am. J. Potato Res..

[44] Xu D., Zhou X.P., Lei C.N., Shang Y., Zhao Y.C., Wang Z.J., Zeng F.K., Liu G. (2020). Development of biscuits and cookies using raw dehydrated potato flour and its nutritional quality and volatile aroma compounds evaluation. J. Food Process. Preserv..

[45] Xinhua News Agency. https://baijiahao.baidu.com/s?id=1819286757953828743&wfr=spider&for=pc.

[46] China Daily Online. https://baijiahao.baidu.com/s?id=1805425455571135505&wfr=spider&for=pc.

[47] Zhao Y.C., Wang X.H., Liao W.J., Xu D., Liu G. (2022). Study on nutritional quality and volatile aroma compounds of the stir-fried shredded potatoes. Am. J. Potato Res..

[48] Wu D., Yang S. (2023). Research on the Brand Building of Potato Seed Tubers in Keshan County Based on SWOT Analysis. Heilongjiang Agric. Sci..

[49] Su W., Wang J. (2019). Potato and food security in China. Am. J. Potato Res..

[50] Feng M. (2020). A Brief Discussion on the Processing and Development of Potato Staple Food Products. Guangdong Seric..

[51] USDA ERS. https://www.ers.usda.gov/data-products/charts-of-note/chart-detail?chartId=99117.

[52] Potato Pro. https://www.potatopro.com/about/french-fries-and-potato-specialties.

[53] Du H.X., Li F.S. (2016). Effects of varying the ratio of cooked to uncooked potato on the microbial fuel cell treatment of common potato waste. Sci. Total Environ..

[54] Bamberg J., Greenway G. (2019). Nutritional and economic prospects for expanded potato outlets. Am. J. Potato Res..

[55] Camire M.E., Kubow S., Donnelly D.J. (2009). Potatoes and human health. Crit. Rev. Food Sci. Nutr..

[56] Morris W.L., Taylor M.A. (2019). Improving flavor to increase consumption. Am. J. Potato Res..

[57] Yagua C.V., Moreira R.G. (2011). Physical and thermal properties of potato chips during vacuum frying. J. Food Eng..

[58] Warning A., Dhall A., Mitrea D., Datta A.K. (2012). Porous media based model for deep-fat vacuum frying potato chips. J. Food Eng..

[59] Willard M. (1993). Potato processing: Past, present and future. Am. Potato J..

[60] Lamberti M., Geiselmann A., Conde-Petit B., Escher F. (2004). Starch transformation and structure development in production and reconstitution of potato flakes. LWT Food Sci. Technol..

[61] Schwartz D., Whistler R.L. (2009). History and future of starch. Starch.

[62] Haverkort A.J., Verhagen A. (2008). Climate change and its repercussions for the potato supply chain. Potato Res..

[63] Zhao J.F., Zhan X., Jiang Y.Q., Xu J.W. (2018). Variations in climatic suitability and planting regionalization for potato in northern China under climate change. PLoS ONE.

[64] Saini R., Kaur S., Aggarwal P., Dhiman A., Suthar P. (2023). Conventional and emerging innovative processing technologies for quality processing of potato and potato-based products: A review. Food Control.

[65] Clark S. (2020). Financial viability of an on-farm processing and retail enterprise: A case study of value-added agriculture in rural Kentucky (USA). Sustainability.

[66] Yan F.B., Wang X.L., Li S.Y., Bao L.X., Li Y.S., Yang W.L., Sui Q.J. (2008). Application of HACCP to the production of fried potato chips. Sci. Technol. Food Ind..

[67] Escuredo O., Meno L., Rodríguez-Flores M.S., Seijo M.C. (2021). Rapid estimation of potato quality parameters by a portable near-infrared spectroscopy device. Sensors.

[68] Zimon D., Madzik P., Domingues P. (2020). Development of key processes along the supply chain by implementing the ISO 22000 standard. Sustainability.

[69] ElMasry G., Cubero S., Moltó E., Blasco J. (2012). In-line sorting of irregular potatoes by using automated computer-based machine vision system. J. Food Eng..

[70] Maurya R., Singh S., Pathak V.K., Dutta M.K. (2021). Computer-aided automatic detection of acrylamide in deep-fried carbohydrate-rich food items using deep learning. Mach. Vision Appl..

[71] Klein O., Nier S., Tamásy C. (2022). Circular agri-food economies: Business models and practices in the potato industry. Sustain. Sci..

[72] Khanal S., Karimi K., Majumdar S., Kumar V., Verma R., Bhatia S.K., Kuca K., Esteban J., Kumar D. (2024). Sustainable utilization and valorization of potato waste: State of the art, challenges, and perspectives. Biomass Convers. Biorefinery.

[73] Kale G., Kijchavengkul T., Auras R., Rubino M., Selke S.E., Singh S.P. (2007). Compostability of bioplastic packaging materials: An overview. Macromol. Biosci..

[74] (2022). CIP Annual Report. https://cipotato.org/?s=Catalyzing+Change%3A+Food+Systems+for+Resilience%2C+Prosperity%2C+and+Health&lang=en.

[75] (2021). CIP Annual Report. https://cipotato.org/?s=From+lab+to+field+to+scale%3A+Demand-driven+solutions+for+food+systems+transformation&lang=en.

[76] (2019). CIP Annual Report. https://cipotato.org/?s=Discovery+to+Impact%3A+Science-based+solutions+for+global+challenges&lang=en.

